# Campbell’s Cow-Bell Method of Casting Plates

**Published:** 1912-01-15

**Authors:** Dayton Dunbar Campbell

**Affiliations:** Kansas City, Mo.


### CAMPBELL'S COW-BELL METHOD OF CASTING PLATES.

BY DAYTON DUNBAR CAMPBELL, KANSAS CITY, MO.

In describing this simple and unique method of casting aluminum plates, we will not go into the question of the superiority of the cast metal denture over one made of vulcanite, but give in a homely way the procedure in detail:

### INVESTMENT MATERIAL.

After taking your impression, coat it with your favorite separating fluid and *when dry rub* well with *soapstone* and pour with an investment compound after the formula originated by Dr. C. C. Allen, which consists of *equal parts* of *Portland cement* and *dental plaster*. This compound is in keeping with the simplicity of the casting machine, and in fact, is cheaper than plaster, sets very fast and is free from shrinkage and expansion, making an ideal compound in bridge work.

It is hardly necessary to say that the plaster and cement must be mixed dry beforehand, and so thoroughly that when patted down with a spatula no particles of plaster are distinctly recognized. To obtain the best results, this material must be worked thicker than plaster.

### SEPARATION.

On separating the impression from the model, if the former when poured is jarred down well, the model will present a finished surface. The part of the *model* the plate is to cover is now *rubbed* well with *soapstone*. This gives the model a metallic luster and a surface very desirable to cast metal against.

### WAX.

The model is now covered with a new, clean piece of common yellow *base-plate wax* and care taken that it is of *even thickness* throughout, and that it has not buckled on the ridge in the median line. The wax is now *trimmed* as you wish the *plate to be*, and care taken to cut it low where the depressor muscles have action, usually in the region of the first bicuspid. The *outer edge* of the *wax* is now *stuck* to the *model* all the way around with a dry, sizzling-hot spatula, held at a right angle with the wax. A *strip* of *wax* 3-16 of an *inch wide* is now cut lengthwise from a sheet of wax and *laid* down *flat* on the *wax* model even with the edge except near the heel of the plate, where it is brought over to the lingual surface. The wax strip is brought as near the top or bottom of the ridge on the palatal or lingual surface as the case will permit, the idea being to do away with as much rubber as is possible. The outer sixteenth of this strip is now made a part of the wax model by thrusting a dry, hot spatula through the strips all the way around *until* the *model* is *felt* beneath. The surface next the outer border of the strip is now filled in with wax and smoothed up with a chip blower and alcohol lamp, care being exercised to leave the inner edge of the strip square. At this point lower the temperature of the wax, which has become soft by working, by dipping model and all in water that is not cold; then, with a dull instrument, *raise* the *inner border* of the *wax strip all the way round.* It should be lifted away most near the canine eminence and the lingual border of the heel. If this is done right, it affords all the attachment necessary. Sometimes it would be impracticable to use the strips on the labial border; it can be done away with altogether, and for the attachment of the rubber, loops of German *silver* or *aluminum can be set in place* on the *wax* and *cast successfully.* Of course, there is no union of the metals, but they are held in place mechanically tight enough to serve their purpose.

If you desire to cast directly on the teeth, the front is left open for the pink rubber, and a space of one-half to one millimeter left between all the teeth. This is imperative, because the contraction of the alloy or metal used in casting the teeth would all be checked or broken off.

The model is now trimmed even with the heel of the plate and a little roll of wax, about the size of a slate pencil and about three inches long, is stuck on the model with a hot spatula, even with and joined to the wax plate. Don't lay this piece of wax over on the wax, as it will not facilitate casting, but will be difficult to remove in the cast.—*Summary.*

